# Characterization of expressed sequence tags from a full-length enriched cDNA library of *Cryptomeria japonica *male strobili

**DOI:** 10.1186/1471-2164-9-383

**Published:** 2008-08-11

**Authors:** Norihiro Futamura, Yasushi Totoki, Atsushi Toyoda, Tomohiro Igasaki, Tokihiko Nanjo, Motoaki Seki, Yoshiyuki Sakaki, Adriano Mari, Kazuo Shinozaki, Kenji Shinohara

**Affiliations:** 1 Department of Molecular and Cell Biology, Forestry and Forest Products Research Institute, Tsukuba, Ibaraki 305-8687, Japan; 2 Computational and Experimental Systems Biology Group, Genomic Sciences Center, RIKEN, Yokohama, Kanagawa 230-0045, Japan; 3 Genome Core Technology Facilities, Genomic Sciences Center, RIKEN Yokohama Institute, Yokohama, Kanagawa 230-0045, Japan; 4 Plant Functional Genomics Research Group, Plant Science Center, RIKEN Yokohama Institute, Yokohama, Kanagawa 230-0045, Japan; 5 Allergy Data Laboratories sc, Via Malipiero 28, 04100 Latina, Italy

## Abstract

**Background:**

*Cryptomeria japonica *D. Don is one of the most commercially important conifers in Japan. However, the allergic disease caused by its pollen is a severe public health problem in Japan. Since large-scale analysis of expressed sequence tags (ESTs) in the male strobili of *C. japonica *should help us to clarify the overall expression of genes during the process of pollen development, we constructed a full-length enriched cDNA library that was derived from male strobili at various developmental stages.

**Results:**

We obtained 36,011 expressed sequence tags (ESTs) from either one or both ends of 19,437 clones derived from the cDNA library of *C. japonica *male strobili at various developmental stages. The 19,437 cDNA clones corresponded to 10,463 transcripts. Approximately 80% of the transcripts resembled ESTs from *Pinus *and *Picea*, while approximately 75% had homologs in *Arabidopsis*. An analysis of homologies between ESTs from *C. japonica *male strobili and known pollen allergens in the Allergome Database revealed that products of 180 transcripts exhibited significant homology. Approximately 2% of the transcripts appeared to encode transcription factors. We identified twelve genes for MADS-box proteins among these transcription factors. The twelve MADS-box genes were classified as *DEF/GLO/GGM13-, AG-, AGL6-, TM3- *and *TM8*-like MIKC^C ^genes and type I MADS-box genes.

**Conclusion:**

Our full-length enriched cDNA library derived from *C. japonica *male strobili provides information on expression of genes during the development of male reproductive organs. We provided potential allergens in *C. japonica*. We also provided new information about transcription factors including MADS-box genes expressed in male strobili of *C. japonica*. Large-scale gene discovery using full-length cDNAs is a valuable tool for studies of gymnosperm species.

## Background

Molecular studies of angiosperm model plants have identified large numbers of genes that are expressed during stamen and pollen development. Genetic analyses have revealed the roles of some of these genes in the specification of stamen identity, the regulation of anther cell division and the differentiation of anthers, the control of male meiosis, the development of pollen, and anther dehiscence [1]. Pollen development in gymnosperms and angiosperms involves several similar developmental and physiological processes [2,3]. Homologs to floral transcription factors in angiosperms have been isolated from gymnosperm strobili and characterized. For example, the B-class MADS-box genes, which in angiosperms determine petal and stamen identities, and C-class genes that control the identities of stamens and carpels have been identified in conifers [4-7]. The Norway spruce gene *DAL1*, belongs to the *AGL6 *subfamily and *DAL10*, belongs to gymnosperm-specific subgroup were identified as other MADS-box genes in conifers [8,9]. The flowering time gene, *SOC1 *and *LEAFY*, and A class gene *APETALA2 *have also been identified [10-13]. However, available information about transcripts that are expressed in the male reproductive structures of the gymnosperms is still limited [14].

The gymnosperm *Cryptomeria *is a monoecious plant that is distributed throughout Japan and in some parts of China. *Cryptomeria japonica *D. Don is widely grown in Japan because of its high productivity and utility. This species comprises 18% of the forests and covers 12% of the landmass of Japan. However, allergic reactions to its pollen have become a severe public health problem in Japan. A recent nationwide epidemiological survey found that at least 13% of the Japanese population suffers from pollinosis due to pollen of *C. japonica *[15].

The male strobili of *C. japonica *develop in axils of small branches near the tips of these branches. The primordia of male strobili are initiated from June to August and first become visible from July to September under natural conditions [16,17]. The development of strobili can also be initiated by treatment with gibberellic acid (GA_3_). The promotion of flower formation by exogenous GA_3 _occurs even in one-year-old seedlings in spite of the fact that formation of strobili usually requires around 20 years after germination under natural conditions [16]. Meiosis of microsporocytes begins in the middle of October, and microspores are formed from late October to late November. Differentiation of generative cells and tube cells occurs in December. The staminate strobili then remain in an arrested state of development until the following March when pollen grains are released. Each male strobilus is oval, pale yellow, and close to 5 mm in length and 2 mm in diameter, and each consists of microsporophylls attached to a main axis. Three to five rounded microsporangia develop on the lower surface of each microsporophyll. Each microsporangium contains 3,000 or more pollen grains, and as many as 400,000 pollen grains may be produced in a single strobilus [16].

Large-scale analysis of expressed sequence tags (ESTs) should help us to clarify the overall expression of genes in the male strobili of *C. japonica*. Some ESTs have already been derived from male strobili and from the pollen of *C. japonica *[18,19]. However, the numbers of ESTs were relatively small, namely, 739 from male strobili and 3,655 from pollen, and most isolated cDNAs were not full-length. In the present study, we constructed a full-length enriched cDNA library using RNA derived from male strobili at various developmental stages, and we obtained more than 30,000 ESTs. We performed sequence-similarity searches using TBLASTX and BLASTX to compare the ESTs from *C. japonica *male strobili to sequences in UniProt, amino acid sequences from *Arabidopsis *and rice (*Oryza sativa *L.), and EST sequences from poplar, spruce and pine. We also identified ESTs that encoded MADS-box genes, which play a variety of important developmental roles in plants. In this report, we discuss both the utility of full-length cDNAs for gene discovery and the characterization of MADS-box genes in *C. japonica*.

## Results

### Construction and quality check of a full-length enriched cDNA library

We constructed a full-length enriched cDNA library from male strobili of *C. japonica *at various stages of development. The mean length of the insert DNA derived from 91 randomly selected clones was approximately 1.6 kbp. We picked up 19,968 clones (clone IDs: CMFL001_A1 through CMFL052_N24) at random and sequenced them from their 5' and 3' ends. In total, 39,936 sequences were obtained and processed for removal of vector sequences, low-quality data and contamination by genomic DNA from *E. coli*. We retained 36,011 ESTs, which encompassed 18,843 sequences of 5' ends and 17,168 sequences of 3' ends derived from 19,437 clones. Each EST consisted of at least 100 contiguous nucleotides with a PHRED score greater than 20 (Table 1; Additional file 1). The average sequenced length of the ESTs was 517 bp. All sequences have been deposited in the dbEST division of DDBJ/EMBL/GenBank. The accession number of each EST is shown in Additional file 1.

**Table 1:** Summary of characteristics of the full-length cDNA library from male strobili of C. japonica

Total sequences	36,011
Number of 5'sequences	18,843
Number of 3' sequences	17,168
Number of cDNA clones	19,437
Number of contigs^a^	7,686
Number of singlets^a^	15,972
Number of unique transcripts^b^	10,463
Number of unique transcripts corresponded to	
1 clone	6,320
2 clones	2,078
3–5 clones	1,686
6–10 clones	353
11–20 clones	21
>21 clones	5

^a ^ESTs were assembled using the PHRAP program.

^b ^Contigs and singlets were grouped using the BLASTN program and then both 5'- and 3'- end sequences derived from the respective same clones were grouped together.

We analyzed the full-length cDNA clones that included sites for the initiation of transcription of mRNA. An additional G at the 5' end is observed in many full-length cDNAs that are obtained by the biotinylated CAP trapper method, which we used in this study [20]. Among the 5' sequence in our cDNA library derived from *C. japonica *male strobili, 16,977 (90%) had G at the 5' end (Table 2). The high percentage of cDNA clones with G at the 5' terminus suggests that the relative level of full-length cDNA clones in our library was high. The second nucleotide showed a strong bias towards purines (89% A or G; Table 2). This observation is consistent with the fact that transcription usually starts at a purine in *Arabidopsis *and rice [21]. Subsequent positions did not show such an extreme bias towards purines. Our results suggest that most full-length cDNAs had one additional G at their 5' termini.

**Table 2:** Base composition at each position in the 5'-end sequences

Position	A	G	C	T	%purine (A+G)
1	505 (2.7%)	16977 (90%)	350 (1.9%)	1011 (5.4%)	93
2	8185 (43%)	8613 (46%)	1066 (5.7%)	979 (5.2%)	89
3	5146 (27%)	5237 (28%)	2978 (16%)	5482 (29%)	55
4	5408 (29%)	5645 (30%)	3545 (19%)	4249 (23%)	59
5	5517 (29%)	5455 (29%)	3225 (17%)	4646 (25%)	58
10	5456 (29%)	3879 (21%)	3849 (20%)	5659 (30%)	50

The occurrence of each base was counted at each position.

To confirm the addition of G at the 5' end of full-length cDNA clones, we compared the 5'-terminal sequences of cDNA clones that encoded Cry j 2 with genomic sequences, including the promoter and coding region of *Cry j 2*, that we determined in a previous study [22]. We found seven clones that encoded Cry j 2 among the 5'-end sequences of 18,843 clones. All seven of them were 5'-extended cDNAs, as compared with three Cry j 2 cDNAs that we generated previously [22]. Five of the seven had one G, one clone had two Gs and one clone had TG at its 5' terminus; these nucleotides were not present in the corresponding region of *Cry j 2 *genes (data not shown). The comparison between these seven cDNAs and *Cry j 2 *genes indicated that full-length cDNAs had one or two additional nucleotides at their 5' termini, with most clones having a single G.

We performed a BLASTX comparison of amino acids encoded by 5'-terminal sequences of 18,843 cDNA clones and proteins from *Arabidopsis*. Our analysis revealed that 9,208 ESTs (48.7%) exhibited strong homology to *Arabidopsis *proteins (*E*-value < 1e-20). Among these ESTs, the starting positions in the alignment of 7,480 clones (81.2%) were upstream of the initiation codon of the corresponding protein. We also performed a BLASTN comparison with nucleotide sequences of protein-coding regions in *Arabidopsis*. We found that 2,131 ESTs (11.5%) derived from 5'-terminal sequences of cDNAs exhibited strong homology (*E*-value < 1e-10) to protein-coding regions in the *Arabidopsis *genome. The starting positions in the alignment of 1,755 ESTs (82.4%) were upstream of the corresponding coding region of *Arabidopsis*. These results suggest that most of our cDNA clones are full-length cDNAs.

### Classification of ESTs

**Figure 1:**
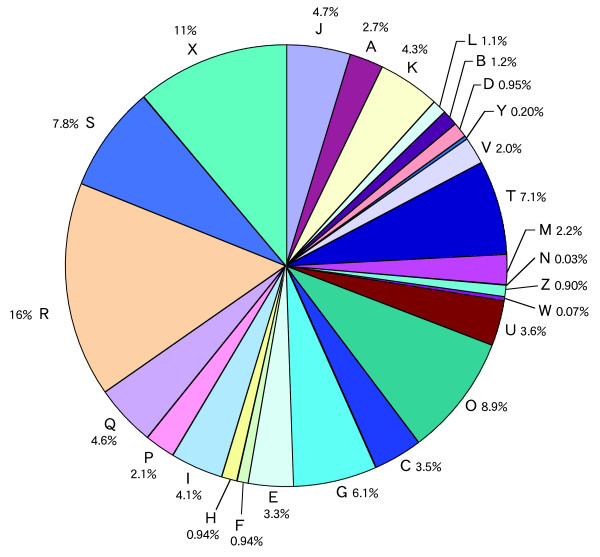
**Functional classification and relative levels, as percentages of unique transcripts in the pool, of ESTs derived from male strobili of *C. japonica***. We assigned 7,369 clusters by reference to databases of KOGs, TWOGs and LSEs using a BLAST-based algorithm (*E*-value ≤ 10^-5^). Designations of functional categories: A, RNA processing and modification; B, chromatin structure and dynamics; C, energy production and conversion; D, cell cycle control and mitosis; E, amino acid transport and metabolism; F, nucleotide transport and metabolism; G, carbohydrate transport and metabolism; H, coenzyme transport and metabolism; I, lipid transport and metabolism; J, translation, ribosomal structure and biogenesis; K, transcription; L, replication and repair; M, cell wall/membrane/envelope biogenesis; N, cell motility; O, post-translational modification, protein turnover and chaperone functions; P, inorganic ion transport and metabolism; Q, secondary metabolites biosynthesis, transport and catabolism; T, signal transduction; U, intracellular trafficking, secretion, and vesicular transport; V, defense mechanisms; Y, nuclear structure; Z, cytoskeleton; R, prediction of general function only; S, function unknown; and X, unassigned.

The assembly of ESTs can be expected to generate an overestimate of the actual number of genes represented since failure of ESTs to assemble can result from alternate splicing, differences in usage of polyadenylation sites, sequence polymorphism, and sequencing errors. Levels of redundancy after EST assembly have been estimated to range from 20% to 22% in previous studies of EST collections [23,24]. To reduce redundancy, we compared all consensus sequences (7,686 tentative contigs and 15,972 singletons) using BLASTN after assembly with PHRAP and then we grouped both 5'- and 3'-end sequences derived from the same respective clones together. Finally, our analysis indicated that ESTs derived from 19,437 clones could be grouped into 10,463 clusters as unique transcripts (Table 1; Additional file 1). The largest cluster contained 35 clones, but only 26 transcripts (0.25%) corresponded to more than 10 clones. We found that 6,320 of the 10,463 transcripts (60.4%) corresponded to only one clone and 2,078 clusters (19.9%) corresponded to two clones. The normalization step appeared to reduce the redundancy of our ESTs. Mitochondrial and chloroplast RNA sequences were not filtered, but they contributed one and three clones to the data set of 19,437 cDNA clones, respectively. No contamination by ribosomal RNA was detected in the data set. Figure 1 shows the functional classification of the putative proteins encoded by our ESTs, which was based on assignments in the COG database [25]. Of the putative proteins derived from individual transcripts, 7,339 (70.1%) were assigned to 26 putative functional categories by BLASTX (E-value < 1e-5; Additional file 2).

### Sequence comparisons with other species

**Figure 2:**
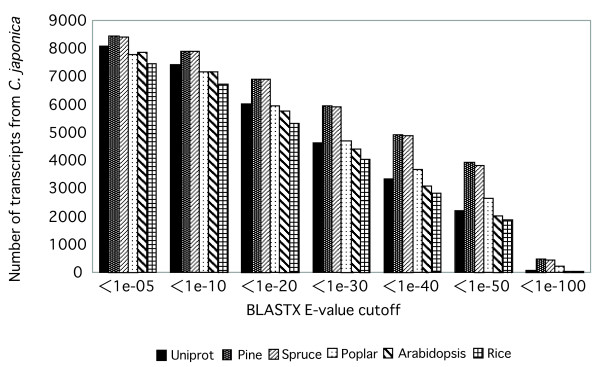
**Sequence similarities**. Numbers of transcript sequences of transcripts from *C. japonica *male strobili similar to sequences in the Uniprot, pine, spruce, poplar, *Arabidopsis*, and rice databases according to BLASTX *E-*value cutoff values.

We found that 8,059 (77.0%) of the 10,463 transcripts encoded peptides that were significantly similar to those in the UniProt database, 7,840 (74.9%) were similar to proteins in *Arabidopsis*, and 7,455 (71.3%) were similar to proteins in *japonica *rice at a BLASTX *E-*value of 1e-5 (Figure 2). When we compared our ESTs with sequences of ESTs from *Pinus, Picea *and *Populus *by TBLASTX, we found that 8,433 (80.6%), 8,400 (80.3%) and 7,763 (74.2%) transcripts included similar ESTs, respectively, at *E-*values of 1e-5. A larger overlap was found in the case of coniferous species (Figure 2). We also compared the 10,463 transcripts examined in the present study with ESTs that had been previously isolated from *C. japonica*, which included 10,328 ESTs derived from cambium and surrounding tissues, 3,677 ESTs from pollen, 3,222 ESTs from male strobili and 1,889 ESTs from other organs [19,26]. Slightly more than half (5,792; 55.4%) of our transcripts did not exhibit any similarity (BLASTN *E*value < 1e-10) to ESTs that had been previously isolated from *C. japonica*. Thus, the ESTs described in the present report contain novel information about protein-coding regions of the *C. japonica *genome.

### Functional analysis

We analyzed conserved domains using Pfam [27] as a database to predict the function of products of *C. japonica *transcripts. Overall, we found that 5,304 (50.7%) of the 10,463 transcripts encoded proteins similar to members of 1,664 Pfam protein families (E-value < 1e-10). Among these Pfam families, 147 families are denoted "DUF, Domain of Unknown Function" and 26 families are denoted "UPF, Uncharacterized Protein Family". In total, products of 4,965 (47.5%) of the transcripts from *C. japonica *male strobili were similar to members of 1,481 Pfam families when DUFs and UPFs were excluded. We found that 4,850 (46.3%) transcripts belonged to only one Pfam family, 433 (4.1%) transcripts belonged to two families, 17 transcripts (0.16%) belonged to three families, and two transcripts (0.02%) belonged to four families (Additional file 2). The most abundant protein families in male strobili of *C. japonica *corresponded to those most strongly represented in the genome of *Arabidopsis *(Table 3). Similar earlier analyses indicated that these domains occur frequently in sugarcane and white spruce also [23,28].

**Table 3:** Occurrence of the 25 most common Pfam domains in the predicted proteins of unique transcripts from male strobili of *C. japonica*

Description of Pfam domain	Pfam accession	Number of *C. japonica *transcripts^a^	Number of genes in *A. thaliana *genome^b^
Protein kinase domain	PF00069	132	789
Cytochrome P450	PF00067	120	242
RNA recognition motif.			
(a.k.a. RRM, RBD, or RNP domain)	PF00076	78	172
NAD-dependent epimerase/dehydratase family	PF01370	54	52
Zinc finger; C3HC4 type (RING finger)	PF00097	48	18
GDSL-like lipase/acylhydrolase	PF00657	44	107
Myb-like DNA-binding domain	PF00249	43	53
WD domain; G-beta repeat	PF00400	41	17
Ras family	PF00071	35	73
UDP-glucuronosyl and UDP-glycosyl transferase	PF00201	35	86
Short-chain dehydrogenase	PF00106	34	55
Alpha/beta hydrolase fold	PF00561	34	37
Sugar (and other) transporter	PF00083	33	55
2OG-Fe(II) oxygenase superfamily	PF03171	33	105
Core histone H2A/H2B/H3/H4	PF00125	32	46
Peroxidase	PF00141	32	82
DnaJ domain	PF00226	32	88
Eukaryotic aspartyl protease	PF00026	31	9
Aldo/keto reductase family	PF00248	30	21
Transferase family	PF02458	28	55
Alcohol dehydrogenase, GroES-like domain	PF08240	28	25
Mitochondrial carrier protein	PF00153	27	55
Ubiquitin-conjugating enzyme	PF00179	27	41
Zinc-binding dehydrogenase	PF00107	26	39
AMP-binding enzyme	PF00501	26	45

^a ^Protein families were identified by BLASTX searches with an E-value < 1e-10 in the Pfam database.

^b ^Protein families were assigned on the basis of data in TAIR.

### Similarity of products of ESTs to stamen- or male gametophyte-specific proteins of *Arabidopsis*

We compared our deduced proteins with stamen and male gametophyte-specific proteins in *Arabidopsis*. Referring to previous studies of *Arabidopsis*, we identified amino acid sequences encoded by 1,145 stamen-specific transcripts and by 1,274 male gametophyte-specific transcripts in the *Arabidopsis *database [29,30]. We found that 754 (65.9%) stamen-specific transcripts and 617 (48.4%) male gametophyte-specific transcripts of *Arabidopsis *resembled 509 and 429 transcripts, respectively, from male strobili of *C. japonica*, at a TBLASTX *E-*value of 1e-10 (Additional files 3 and 4). Table 4 shows the Pfam domains that are found in stamen- and male gametophyte-specific genes of *Arabidopsis *and the numbers of transcripts that correspond to the families of genes in male strobili of *C. japonica*. Our results suggest that homologs of stamen- and pollen-specific transcripts of *Arabidopsis *are expressed in the male strobili of *C. japonica*.

**Table 4:** Pfam domains found in transcripts of both *A. thaliana *stamen- or male gametophyte-specific genes and in transcripts from male strobili of *C. japonica*

Description of Pfam domain	Pfam accession number	Number of transcripts in *C. japonica *male strobili	Number of *A. thaliana *stamen- specific transcripts^a^	Number of *A. thaliana *male gametophyte-specific transcripts^b^
Protein kinase domain	PF00069	132	35	39
Plant invertase/pectin methylesterase inhibitor	PF04043	12	22	15
Protein tyrosine kinase	PF07714	19	16	12
GDSL-like lipase/acylhydrolase	PF00657	44	15	4
Pectinesterase	PF01095	14	15	11
ABC transporter	PF00005	20	13	5
Cytochrome P450	PF00067	120	13	3
Glycosyl hydrolase family 28	PF00295	10	12	11
Sodium/hydrogen exchanger family	PF00999	6	9	10
ABC-2 type transporter	PF01061	8	9	3
Oleosin	PF01277	4	9	4
No apical meristem (NAM) protein	PF02365	12	9	2
RNA recognition motif.				
(a.k.a. RRM, RBD, or RNP domain)	PF00076	78	8	8
Sugar (and other) transporter	PF00083	33	8	3
Calcineurin-like phosphoesterase	PF00149	25	8	6
E1-E2 ATPase	PF00122	4	7	4
Multicopper oxidase	PF00394	4	7	4
Haloacid dehalogenase-like hydrolase	PF00702	19	7	3
Multicopper oxidase	PF07731	14	7	4
Multicopper oxidase	PF07732	17	7	4
Glycosyl hydrolase family 1	PF00232	20	6	4
Pectate lyase	PF00544	5	6	3
FAD-binding domain	PF01565	6	6	2
Galactose-binding lectin domain	PF02140	5	6	4
MtN3/saliva family	PF03083	14	6	1

^a ^1,145 transcripts were picked up on the basis of previous report by Wellmer et al. (2004) [29].

^b ^1,274 transcripts were picked up on the basis of previous report by Honys and Twell (2004) [30].

### Similarity of the deduced proteins to pollen allergens

We compared the peptides encoded by all the ESTs with known plant allergens in Allergome [31]. Table 5 shows a list of pollen allergens that are similar to the deduced proteins encoded by ESTs derived from *C. japonica *male strobili. Proteins encoded by 180 transcripts (1.7%) exhibited partial sequence homology to plant allergens, which included known allergens of *C. japonica*. This result suggests the possibility that unidentified allergens in *C. japonica *might exist in our EST collection.

**Table 5:** Products of ESTs that resemble pollen allergens

Accession No.^a^	Allergen	Species	Putative product	E-value^b^	No.^c^
BY894724	Cry j 1	*Cryptomeria japonica *(Sugi)	Pectate lyase	1E-127	6
BY895894	Cry j 2	*Cryptomeria japonica *(Sugi)	Polymethylgalacturonase	1E-119	10
BY891770	Cry j 3.8	*Cryptomeria japonica *(Sugi)	PR-5 protein	3E-98	16
BY896705	CJP-4	*Cryptomeria japonica *(Sugi)	Class IV chitinase	1E-112	4
BY888350	CJP-6	*Cryptomeria japonica *(Sugi)	Isoflavone reductase family	4E-87	7
BY894232	Jun o 4	*Juniperus oxycedrus *L. (Prickly juniper)	Calcium-binding protein	2E-67	16
BY912188	Amb a 3	*Ambrosia artemisiifolia *L. (Ragweed)	Plastocyanin-like protein	2E-8	2
BY881070	Cat r 1	*Catharanthus roseus *(L.) G. Don (Madagascar periwinkle)	Cyclophilin	9E-79	10
BY882008	Che a 1	*Chenopodium album *L. (Lamb's-quarters)	Trypsin inhibitor	3E-28	1
BY911759	Cor a 1.04	*Corylus avellana *L. (Hazel)	PR-10 protein	2E-14	1
BY896550	Cor a 10	*Corylus avellana *L. (Hazel)	Luminal-binding protein	1E-107	10
BY882008	Cro s 1	*Crocus sativus *L. (Saffron)	LAT52 protein	3E-13	3
BY883628	Cyn d 22	*Cynodon dactylon *(L.) Pers. (Bermuda grass)	Enolase	9E-25	1
BY893554	Cyn d 24	*Cynodon dactylon *(L.) Pers. (Bermuda grass)	PR-1 protein	2E-33	3
BY895449	Hum j Profilin	*Humulus japonicus *Siebold & Zucc. (Japanese hop)	Profilin	6E-63	3
BY899168	Hum j 1	*Humulus japonicus *Siebold & Zucc. (Japanese hop)	Uncharacterized protein	1E-10	3
BY892250	Lol p 1	*Lolium perenne *L. (perennial ryegrass)	Expansin	6E-14	11
BY896420	Ole e 5	*Olea europaea *L. (Olive)	Superoxide dimutase	7E-66	5
BY894301	Ole e 9	*Olea europaea *L. (Olive)	β-1,3-glucanase	2E-46	10
BY905708	Ole e 10	*Olea europaea *L. (Olive)	Glycosyl hydrolase	4E-23	3
BY889873	Sal k 1.03	*Salsola kali *L. (Russian-thistle)	pectin esterase	2E-35	2
BY911213	Sal k 2	*Salsola kali *L. (Russian-thistle)	Protein kinase	6E-47	53

^a ^The accession number of an EST that exhibited strongest similarity.

^b ^The *E-*value of an EST that exhibited strongest similarity.

^c ^Total number of transcripts whose products were similar to the respective allergens (BLASTX score > 50 and *E-*value <10^-7^).

### Families of putative transcription factors

Transcription factors play important roles in the formation of floral tissues throughout organogenesis. To identify transcription factors, we first identified 37 Pfam domains that encode transcription factors by comparing data in the AtTFDB (*Arabidopsis thaliana *transcription factor database) [32] with the domain annotations of *Arabidopsis *proteins. As a result of a search for sequence similarity between ESTs from *C. japonica *male strobili and the 37 identified Pfam domains, we identified a total of 207 (2.0%) unique transcripts that encoded putative transcription factors, which could be assigned to 29 protein families (Table 6). Transcripts whose products resembled the C3HC4 zinc finger domain, MYB-like transcription factor, and AP2 were abundant in the male strobili of *C. japonica*.

**Table 6:** Identification of transcripts encoding putative transcription factors in male strobilus of *C. japonica*

Description of Pfam domains	Pfam accession	Number of *C. japonica *male strobilus transcripts
Zinc finger, C3HC4 type (RING finger)	PF00097	48
Myb-like DNA-binding domain	PF00249	43
AP2 domain	PF00847	18
No apical meristem (NAM) protein; NAC domain	PF02365	12
Homeobox domain	PF00046	11
PHD-finger	PF00628	11
SRF-type transcription factor; MADS box	PF00319	10
Histone-like transcription factor (CBF/NF-Y) and archaeal histone	PF00808	7
Helix-loop-helix DNA-binding domain	PF00010	6
HSF-type DNA-binding	PF00447	6
B-box zinc finger	PF00643	6
Dof domain, zinc finger	PF02701	4
WRKY DNA-binding domain	PF03106	3
Response regulator receiver domain	PF00072	2
bZIP transcription factor	PF00170	2
GATA zinc finger	PF00320	2
B3 DNA binding domain; ABI3/VP1 transcription factor	PF02362	2
SBP domain	PF03110	2
GRAS family transcription factor	PF03514	2
ZF-HD protein dimerisation region	PF04770	2
CCT motif; CO-like protein	PF06203	2
Auxin response factor	PF06507	2
ARID/BRIGHT DNA binding domain	PF01388	1
CCAAT-binding transcription factor (CBF-B/NF-YA) subunit B	PF02045	1
TCP family transcription factor	PF03634	1
CG-1 domain; CAMTA protein	PF03859	1
YABBY protein	PF04690	1
Plant protein of unknown function; BZR1/LAT61 family	PF05687	1
Whirly transcription factor	PF08536	1

### Identification and phylogenetic analysis of MADS-box genes

**Figure 3:**
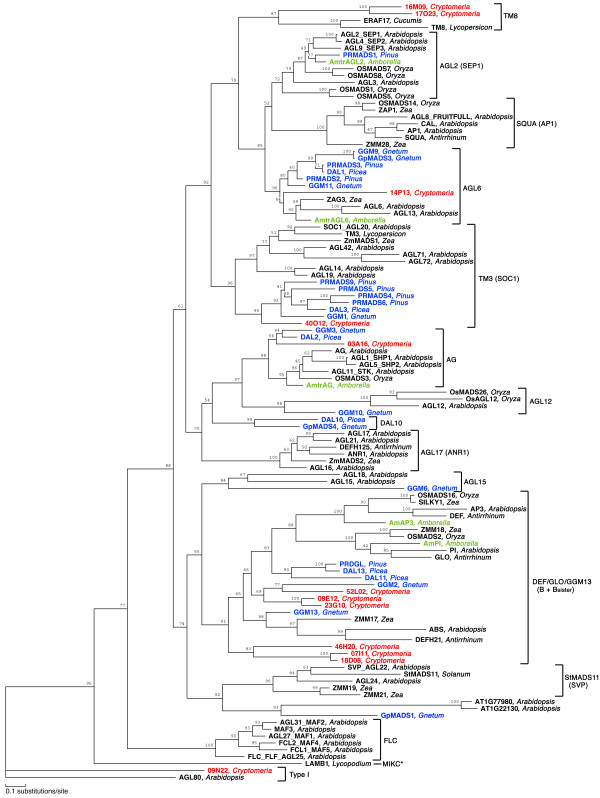
**A phylogenetic tree based on MADS, I- and K-domains of deduced proteins**. The local bootstrap probability is shown on branches where available. This tree is an unrooted tree. MIKC^C^-type genes are divided into 13 subfamilies, including gymnosperm-specific *DAL10*-like subfamily, on the basis of this tree. The genus from which each transcript was isolated is indicated after the deduced protein name. Deduced proteins from *C. japonica *that were identified in the present study are indicated in red. Deduced proteins from gymnosperms other than *C. japonica *are indicated in blue, and deduced proteins from basal angiosperms are indicated in green.

Among the putative transcription factors, ten transcripts encoded MADS-box proteins (Table 6). In addition, we found another two transcripts whose products were similar to MADS-box proteins with *E-*values < 1e-8. We determined the entire sequence of representative cDNA derived from each transcript (accession numbers AB359027 through AB359038). Figure 3 shows the phylogenetic tree constructed from an alignment of the sequences of MADS, I-, and K-domains. One cDNA clone (CMFL009_N22) did not encode a K-domain and it was classified as the cDNA of a type I MADS-box gene. Six of twelve *C. japonica *MADS-box genes were classified as *DEF/GLO/GGM13*-like genes and two genes were categorized as *TM8*-like genes. The other three genes were categorized as *AG-, AGL6*-, and *TM3*-like genes, respectively (Figure 3).

## Discussion

The genomes of gymnosperm species are usually large and replete with highly repetitive sequences. The haploid DNA content of *C. japonica *was estimated to correspond to 11 pg of DNA [33]. This value is about half of the mean value for Pinaceae species [34], but about eighty times that of the *Arabidopsis *genome. EST analysis is a low-cost approach to characterization of the coding component of the genome. ESTs derived from several tissues of *C. japonica *were analyzed previously [18,19,35]. However, most of the previously analyzed ESTs were not full-length. Full-length cDNAs are essential for the determination of sites of initiation of transcription and the functional analysis of genes [36]. In the present study, we constructed a full-length enriched cDNA library from *C. japonica *male strobili at various stages of development. To our knowledge, this is the first report of a full-length enriched cDNA library from a gymnosperm. In fact, the information of 6,464 Sitka spruce full-length cDNA sequences were registered in GenBank (accession numbers EF081469 to EF087932), but it has not been published. These full-length cDNAs should be a valuable tool for gene discovery and analysis in gymnosperms. We found that 90% of our cDNA clones included G at the 5' ends (Table 2). The G at the 5' end of full-length cDNA can be explained by the addition of C to the 3' end of full-length first-strand cDNA in a non-template-directed manner by the reverse transcriptase [37]. Our observation of bias towards G at the 5' end of cDNAs is consistent with the addition of C to the 3' end of full-length cDNAs. The sequences of 5' ends imply that our cDNA library had been enriched for full-length cDNA clones. We also found that the second nucleotide of most cDNAs at their 5' termini was a purine nucleotide, which is common at sites of initiation of transcription in *Arabidopsis *and rice [21]. This result suggests that a purine nucleotide is common at sites of initiation of transcription in *C. japonica *as well as *Arabidopsis *and rice. However, further studies about the comparison between genome sequences and the full-length cDNAs are needed to identify the sequence characteristics around the transcription start site in *C. japonica*.

We isolated 36,011 ESTs from either one or both ends of 19,437 cDNA clones. These ESTs were clustered into 23,658 consensus sequences (7,686 tentative contigs and 15,972 singletons) by PHRAP. The redundancy is extremely low compared with assemblies of ESTs derived from other conifers. In previous studies, the assembly of 49,101 ESTs derived from 16 cDNA libraries from different tissues of white spruce resulted in 16,578 consensus sequences [28], and that of 59,797 ESTs from wood-forming tissues of loblolly pine represented 20,377 consensus sequences [38]. The normalization step in the generation of our cDNA library contributed the high rate of gene discovery. We obtained 10,463 transcripts after a round-robin BLASTN search and clustering of 5'- and 3'-end sequences derived from the same respective clones. We found that 1,317 transcripts (12.6%) did not exhibit any similarity in terms of predicted amino acid sequences to those encoded by *Arabidopsis *and rice genomes or to those derived from *Pinus, Picea *and *Populus *ESTs using BLASTX and TBLASTX (*E-*value > 1e-5). These results indicate that the ESTs generated in the present study have added to the known complement of gymnosperm transcripts.

We classified and annotated the transcripts isolated in this study (Fig. 1; Table 3; Additional file 2). In our functional classification, categories with no concrete assignment, such as "prediction of general function only", "function unknown" and "unassigned", accounted for a large fraction of transcripts (Fig. 1). Among assigned pollen transcripts of *C. japonica*, close to 31% of the predicted proteins are involved in the synthesis and modification of proteins, which are categorized as J and O [19]. However, the percentage in those categories was only approximately 14% in the present study. The difference might be due to the large population of transcripts in the present study and/or to the specific characteristics of the male strobili of *C. japonica*. Table 3 shows the functional annotation of the most abundant families identified in this study. Protein kinase, cytochrome P450, and the RNA recognition motif were the most frequent domains. These domains are also present at relatively high levels in deduced products of the genome of *Arabidopsis*. We also found that the numbers of transcripts that encoded certain protein families or domains, such as NAD-dependent epimerase/dehydratase, the C3HC4 type zinc finger, the WD domain, aspartyl proteases and aldo/keto reductases, were larger than those that encoded the corresponding protein families or domains in the *Arabidopsis *genome. In general, the degree of complexity of multigene families seems to be correlated with the size of the plant genome. Southern hybridization patterns and comparative sequence analysis of conserved ortholog sets suggested that genomes of gymnosperms include complex families of genes [39,40]. Our results suggest the increased complexity of gene families in *C. japonica *as compared to *Arabidopsis*. Since the number of cDNA clones in the present study was insufficient to allow us to prove this hypothesis and since the complete sequence of each clone has not been determined, further studies are needed to clarify the complexity of gene families in *C. japonica*.

We also searched potential candidates for novel allergens in *C. japonica*. In previous study, we analyzed similarity of the deduced proteins encoded by ESTs derived from *C. japonica *pollen and known plant allergens, and found cDNA clones that encoded proteins similar to six types of pollen allergens, eight types of food allergens and three types of latex allergens [19]. In the present study, we found 180 transcripts that encoded proteins similar to 22 pollen allergens including all of five known allergens in *C. japonica *(Table 5). The major allergens in *C. japonica *are known to have similarity to pollen allergens of other plants [19]. The existence of numerous allergens in *C. japonica *pollen has been suggested but only a few antigens have been identified. These newly deduced proteins in *C. japonica *are potential candidates for novel plant allergens.

In this study, we identified 207 transcripts that encoded Pfam domains of transcription factors, including those encoded by MADS-box genes. In plants, MADS-box genes are involved in various aspects of vegetative and reproductive growth, including the morphogenesis of flowers. Plant MADS-box genes are classified into types I and II [41]. Most of plant type II genes have three more domains than type I, namely, an intervening (I) domain, a keratin-like (K) domain, and a C-terminal (C) domain. The plant type II genes are divided into two types based on the intron structure, namely, MIKC^C^- and MIKC*-type genes [42]. The MIKC^C^-type genes can be subdivided into several well-defined gene clades, known as 'subfamilies' [43]. We found twelve MADS-box genes derived from transcripts from male strobili of *C. japonica*, one of which was a type I gene while the other eleven were MIKC^C^-type genes. The MIKC^C^-type genes in *C. japonica *could be subdivided into five subfamilies: *DEF/GLO/GGM13-, TM8-, AG-, AGL6-*, and *TM3*-like genes. To our knowledge, this is the first report to identify type I genes and *TM8*-like genes in a gymnosperm. Functional characterization of type I genes in plants has been very limited to date. The type I gene in *C. japonica *(CMFL_009_N22) was most similar to *AGL80*, which is required for development of the central cell of the female gametophyte [44]. The functions of type I genes in reproductive organs in *C. japonica *remain to be determined.

The MADS-box gene family has been subjected to a model of birth-and death evolution [41]. *TM8*-like genes have been found in rosids (Cucurbitaceae) and asterids (Solanaceae) but not in *Arabidopsis *[43]. A recent review noted that *TM8*-like genes exist in the basalmost angiosperms (Amborellaceae) and in magnoliids (Lauraceae) [45]. We found evidence for at least one *TM8*-like gene in *Cryptomeria *(Cupressaceae sensu lato). None has been reported in Pinaceae to date, to our knowledge. These results suggest that *TM8*-like genes were established in the common ancestor of angiosperms and gymnosperms and that they have been lost relatively recently in some lineages. It seems likely that the rate of birth-and-death evolution of *TM8*-like genes has been high.

We identified six *DEF/GLO/GGM13-*like genes in this study. *DEF*- and *GLO*-like genes are known as B-class MADS genes, which are key regulators of petal and stamen specification in model species of angiosperms, such as *Arabidopsis thaliana, Antirrhinum majus*, and *Petunia hybrida. GGM13-*like genes are the sister lineage of the B-class genes and are, hence, also known as Bsister (Bs) genes [46]. Bs genes are expressed mainly in female reproductive organs and a member of the Bs gene family is involved in development of the seed coat [47]. B-class and Bs genes were treated as one clade in the present study. Three genes (CMFL_009_E12, CMFL_023_G10, CMFL_052_L02) seem to be ancestral *DEF*- and *GLO*-like genes, while the other three genes (CMFL_007_I11, CMFL_018_D08, CMFL_046_H20) seem to constitute a sister clade of B-class and Bs genes. Several gene duplications appear to have occurred in the lineage to *Cryptomeria *after it diverged from other conifers.

The phylogenetic analysis of MADS-box genes in the present study has implications for the evolution of gymnosperms. The phylogenetic tree of *TM3*-like genes indicates that the Gnetales and the Pinaceae are nested as sister groups and that *Cryptomeria *is a sister of the Gnetales plus Pinaceae clade. A similar result was obtained for *AG*-like genes, but the bootstrap support was not very strong. Our data support those in prior multigene phylogenetic studies that suggested that all conifers, with the exception of Pinaceae, are a sister clade of the Gnetales plus Pinaceae clade [48,49]. The evolution and divergence of the MADS-box family of genes has been studied extensively. However, most of the studied MADS-box genes in gymnosperms were derived from *Gnetum, Pinus*, and *Picea *[50,51]. The identification and characterization of MADS-box genes in *Cryptomeria *and other gymnosperms should help us to understand the evolution of the structure of MADS-box genes and their roles in reproductive development.

## Conclusion

We established a full-length enriched cDNA library using RNA derived from male strobili at various developmental stages, and we obtained 36,011 ESTs that was grouped into 10,463 clusters as unique transcripts. These full-length cDNAs provide information on expression of genes during the development of male reproductive organs. Our full-length enriched cDNA library will be useful for large-scale gene discovery for studies of gymnosperm species.

## Methods

### Plant Materials

Male strobili of *C. japonica *were collected at seven-days intervals from mid August to mid November of 2003 and 2004. All the samples were immediately frozen in liquid nitrogen and stored at -80°C until use. RNA was extracted from each set of strobili, and a total RNA mixture derived from all the samples was used for construction of a full-length enriched cDNA library.

### RNA isolation

Total RNA was isolated by a previously described method [52] with slight modifications, using the SV Total RNA Isolating System (Promega, Madison WI). Frozen male strobili were powdered with Multi-beads shocker^® ^(Yasui Kikai, Osaka, Japan). Then we added 7.5 ml of a solution of 100 mM Tris-HCl (pH 9.5), 20 mM EDTA, 1.4 M NaCl, 2% (w/v) polyvinylpyrrolidinone, 5% (w/v) β-mercaptoethanol and 0.5 mg/ml spermidine per gram of powdered male strobili with through mixing. After incubation of the mixture at 65°C for 5 min, RNA was extracted twice with an equal volume of a mixture of chloroform and isoamyl alcohol (24:1, v/v) and centrifugation at 15,000 × *g *for 20 min. One-fourth volume of 10 M LiCl was added to the final aqueous phase. Total RNA was allowed to precipitate for 2 h and collected by centrifugation at 15,000 × *g *for 30 min. The pelleted RNA was dissolved in SV RNA Lysis Buffer (Promega) and purified with the SV Total RNA Isolating System according to the protocol provided by the manufacturer. Poly(A)^+ ^RNA was prepared with a μMACS™ mRNA Isolation Kit (Miltenyi Biotec, Bergisch Gladbach, Germany) according to the protocol provided by the manufacturer.

### Construction of a full-length enriched cDNA library and DNA sequencing

A full-length enriched cDNA library was generated from poly(A)^+ ^RNA by the biotinylated CAP trapper method using trehalose-thermoactivated reverse transcriptase as described previously [36,53]. The resultant cDNAs were normalized, inserted into λ-FLC-III vectors and packaged [54,55]. The packaged DNA produced approximately 1.1 × 10^6 ^plaques and the original phage library was amplified once. Part of the one-round-amplified phage library was excised *in vivo *and resultant plasmids were used to transform *E. coli *DH10B. Colonies of bacteria were picked with picking machines (Q-bot; Genetix, New Milton, UK) and transferred to 384-microwell plates. DNA templates corresponding to cDNA inserts were prepared from glycerol stocks by the rolling circle amplification (RCA) method with a TempliPhi™ DNA Amplification Kit (GE Healthcare Bio-Sciences Corp., Piscataway, NJ, USA). Products of RCA were sequenced with BigDye^® ^Terminator v3.1 Cycle Sequencing Kits (Applied Biosystems, Foster City, CA) and automatic sequencers (ABI 3700; Applied Biosystems). The M13-21 primer (5'-TGT AAA ACG ACG GCC AGT-3') was used for forward sequencing and the 1233 primer (5'-AGC GGA TAA CAA TTT CAC ACA GGA-3') was used for reverse sequencing. Sequences were quality-trimmed by reference to the high-quality (PHRED 20 or better) contiguous region, as determined with PHRED software [56] and then vector and poly(A) regions were removed. Sequences of less than 100 nucleotides after trimming were discarded. Contaminating regions of *E. coli *genome were identified with BLASTN [57], and ESTs with an *E-*value < 1e-10 were excluded. The remaining sequences were used for further analysis.

We searched for the nucleotide and amino acid sequences in TAIR (The Arabidopsis Information Resources; genome release version 6.0) [58] homologous to nucleotide and predicted amino acid sequences encoded by the 5'-end sequences of ESTs using the BLASTN and BLASTX program. We also searched for sequence homologies between the 5'-end sequences of ESTs and the cDNA and promoter region of *Cry j 2 *using the BLASTN program. Assessment of possible contamination by chloroplast and mitochondrial genomes and by ribosomal RNAs was performed with the BLASTN program (*E*-value < 1e-10). We used the chloroplast and mitochondrial genomes of *Arabidopsis thaliana *and genes for ribosomal RNAs from *A. thaliana, Populus *species, *Platanus occidentalis *and *Zea mays *as query sequences.

### Grouping of ESTs

Related cDNA sequences from both 5' and 3' ends were grouped as contig sequences with the PHRAP program according to the following criteria: -penalty -5; -minmatch 300; -minscore 300; and -trim_qual 20 [59]. After grouping ESTs as contigs, we used the BLASTN program to compare contig sequences. When contig sequences overlapped by more than 200 nucleotides and were more than 99% homologous in the overlapping regions and, in addition, when the overlap began within 5 bp from the ends of the contig sequences, we grouped the contig sequences together. We performed BLASTN searches separately for the EST sequences of between 100 and 200 bp and the resultant contig sequences. When an EST sequence and a contig were more than 98% homologous and the length of non-overlapping sequence in the EST sequence was less than 10 bp and, in addition, the overlap began within 5 bp from the end of the EST sequence or the contig, we grouped the EST sequence and the contig together. Finally both 5'- and 3'-end sequences derived from the same clone were grouped together.

### Functional classification and annotation of ESTs

We categorized ESTs on the basis of the putative functions of encoded products using a database of clusters of orthologous groups from seven eukaryotic genomes (COG) [25]. We used databases of proteins from more than three species (KOGs), proteins from two species (TWOGs) and lineage-specific expansion groups (LSEs). We compared the results of BLASTX analysis of each sequence in each cluster and adopted the functional category with the highest score.

We performed similarity searches with the BLASTX and TBLASTX programs. We used RefSeq of *Arabidopsis *from the National Center for Biotechnology Information [60], the database of the Knowledge-based Oryza Molecular Biological Encyclopedia [61] and UniProtKB/TrEMBL Release 33.3 [62] as protein databases, and we used *Pinus *Gene Index Release 6.0, Spruce Gene Index Release 2.0, and Poplar Gene Index Release 3.0 from the TIGR Gene Indices [63] as EST databases. Sequences of stamen- and pollen-specific transcripts of *Arabidopsis *were obtained from previous studies [29,30]. Similarities to ESTs that had previously been derived from *C. japonica *were determined with the BLASTN program. Similarities to known plant allergens were determined by BLASTX comparison with proteins in Allergome database [31]. We annotated protein families using a list of Pfam domain sequence (Pfam-A.fasta; release 21.0) [27] using the BLASTX program and an *E-*value < 1e-10. We obtained domain annotations of *Arabidopsis thaliana *proteins from the TAIR website [58] and selected Pfam families with an *E-*value < 1e-10.

### Phylogenetic analysis

To reconstruct a phylogenetic tree of MADS-box genes, we obtained amino acid sequences from EMBL/DDBJ/GenBank DNA databases and aligned them using the Clustal W program [64]. The alignments of sequences of MADS, intervening (I-) and keratin-like (K-) domains were edited manually using the MacClade program [65]. Maximum likelihood distances were calculated with the NJdist and ProtML programs in MOLPHY [66]. A neighbor-joining (NJ) tree was obtained with NJdist, and was based on ML distances in the JTT model [67]. This tree was used as the starting tree for a local rearrangement search with the ProtML program [66]. The local bootstrap probability of each branch was estimated by the resampling-of-estimated-log-likelihood (RELL) method [68].

## Authors' contributions

NF designed this study, prepared plant materials and RNA, managed the construction of a full-length enriched cDNA library, performed similarity searches and phylogenetic analysis, and drafted the manuscript. YT and AT did the sequencing work, the gene clustering, the gene annotation and submitted the data to DDBJ. AM provided the sequences and data on allergens derived from the Allergome Database. TI, TN, MS, YS, KaS and KeS participated in the design and coordination of the study.

## Supplementary Material

Additional File 1 **Annotation and classification of all ESTs from male strobili of *Cryptomeria japonica***. Annotation and classification of all ESTs from male strobili of *Cryptomeria japonica*. This file shows that the list of DDBJ accession numbers in dbEST, the result of BLASTX against Uniprot TrEMBL, AtCDS and rice cDNA clones, and the functional classification using COG database for ESTs derived from the full-length enriched cDNA library of *C. japonica *male strobili. Click here for file

Additional File 2 **Classification and annotation of unique transcripts from male strobili of *Cryptomeria japonica***. Classification and annotation of unique transcripts from male strobili of *Cryptomeria japonica*. This file shows that the COG classification and Pfam families of unique transcripts from male strobili of *C. japonica*. Click here for file

Additional File 3 **List of stamen-specific transcripts from *Arabidopsis *and corresponding ESTs from male strobili of *Cryptomeria japonica***. List of stamen-specific transcripts from *Arabidopsis *and corresponding ESTs from male strobili of *Cryptomeria japonica *Description of data: This file shows that the list of stamen-specific transcripts from *Arabidopsis *and their homologs derived from ESTs from male strobili of *C. japonica*. Click here for file

Additional File 4 **List of male gametophyte-specific transcripts from *Arabidopsis *and corresponding ESTs from male strobili of *Cryptomeria japonica***. List of male gametophyte-specific transcripts from *Arabidopsis *and corresponding ESTs from male strobili of *Cryptomeria japonica*. This file shows that the list of male gametophyte-specific transcripts from *Arabidopsis *and their homologs derived from ESTs from male strobili of *C. japonica*. Click here for file
